# Use of Micro/Nanorobots In Vivo for the Eradication of Bacterial Biofilm: A Review of Challenges and Strategies

**DOI:** 10.3390/nano16110642

**Published:** 2026-05-22

**Authors:** Ondrej Musil, Karel Klíma

**Affiliations:** Department of Oral and Maxillofacial Surgery, 1st Faculty of Medicine, General University Hospital in Prague, Charles University, 12808 Prague, Czech Republic

**Keywords:** nanomedicine, micro/nanorobots, micro/nanorobotics, bacterial infection, biofilm, in vivo

## Abstract

The term bacterial biofilm refers to a complex community of microorganisms embedded within a self-produced matrix of extracellular polymeric substances. This structural organization creates an environment that, when present in an infectious context within a living organism, limits the effectiveness of conventional antibiotic therapy. Consequently, such conditions substantially promote the development of antibiotic resistance. The decline in the discovery of novel antibiotic agents, coupled with a concurrent increase in the prevalence of multidrug-resistant microorganisms, has intensified the search for alternative strategies to combat such infections. At the same time, advances in nanoscience have stimulated substantial research into the use of micro/nanorobots for the eradication of bacterial biofilms. These devices, engineered at the micro- to nanoscale, are capable of targeted intervention in otherwise inaccessible sites. However, the development of such “microscopic therapeutic agents” is still at an early stage. To date, the vast majority of available data has been derived from in vitro studies, while evidence regarding their feasibility, safety, and therapeutic effects in living organisms remains limited. This review discusses their antimicrobial mechanisms and critically evaluates the current evidence concerning their in vivo applications.

## 1. Introduction

Bacterial infections have historically exerted a substantial impact on average life expectancy and have been responsible for significant morbidity and mortality, even within industrialized nations [1]. Approximately one quarter of children died before the age of fifteen, with estimates suggesting that at least half of these deaths were attributable to infectious diseases [2]. With the advent of the antibiotic era, this pattern shifted, leading to a decline in infection-related morbidity and mortality [3]. From that point onward, such infections have been mainly life-threatening for older adults, people with cancer, transplant recipients, surgical patients, individuals on immunosuppressive treatment, and other high-risk groups. However, even during the so-called Golden Age of antibiotics, which refers to the period from the 1930s to the 1960s when the discovery of new antibiotics peaked, antibiotic resistant bacterial strains were already being observed [4]. Due to the ongoing decline in the discovery of new antimicrobial agents, together with the widespread misuse and overuse of antibiotics in modern medicine and industry, there is a significant risk of a regression to the previously described state prior to the introduction of antibiotics. Current projections indicate that by 2050, infections caused by antibiotic-resistant bacteria could become the leading global cause of mortality, resulting in approximately 10 million deaths annually [5]. Bacterial biofilm-associated infections are particularly challenging to manage due to their frequently chronic nature and intricate structural organization. Consequently, often inadequate antibiotic therapy, coupled with the efficient transfer of resistance genes within the biofilm matrix, substantially contributes to the emergence and proliferation of antibiotic resistance [6].

In recent years, advances in nanoscience have enabled a novel approach to addressing such infections through the use of micro/nanorobots. These devices, at micro- to nanoscale dimensions, represent a promising technology capable of autonomous or externally actuated motion, allowing them to navigate confined environments, deliver therapeutic cargo to targeted sites, and manipulate microorganisms [7]. Their capacity to carry out essential functions at both the cellular and subcellular scale is enabled by their intrinsic controllability, including directed movement, selective target identification, and precisely triggered functional activity [8]. Furthermore, these systems benefit from intentionally engineered stimuli-responsive properties, which facilitate adaptive transformations required for the performance of specific functions and designated tasks [9]. Consequently, such features enable active targeting and intelligent therapeutic action, thereby minimizing side effects while improving treatment efficacy [10]. The above-described features represent essential properties distinguishing micro/nanorobots from passive nanoparticles, as well as theoretically make them promising candidates for future implementation in medical practice.

This review provides a concise overview of bacterial biofilm composition, structural organization, and current treatment strategies, along with their limitations and emerging scientific perspectives. Antimicrobial approaches and the mechanisms of micro/nanorobots are highlighted, with particular emphasis on cargo delivery, reactive oxygen and nitrogen species (RONS) generation, physical disruption, and their synergistic applications. Finally, the existing literature on in vivo eradication of bacterial biofilms using micro/nanorobots is systematically examined. This review provides a perspective that differs from existing reviews in the field, as it was prepared from the standpoint of potential end users and biomedical researchers rather than primarily from a nanoscience-oriented perspective. By focusing on the translational and practical relevance of these systems, especially through the discussion of available in vivo research findings, this review aims to complement the current literature and provide a broader perspective for future potential clinical applicability of micro/nanorobotic systems.

## 2. Biofilms in the Human Body

The first person to observe bacterial communities attached to surfaces was Anthony van Leeuwenhoek. However, the term “biofilm” was described later by Costerton et al. 1978 [11].

Biofilms are highly organized, surface-associated microbial communities embedded within a self-produced extracellular polymeric substance (EPS) matrix. In the human host, biofilms are implicated in both commensal and pathogenic contexts, contributing significantly to the persistence and chronicity of a wide spectrum of infections. Microorganisms within biofilms exhibit phenotypic heterogeneity and enhanced resistance to host immune defenses and conventional antimicrobial agents, largely due to restricted diffusion, altered microenvironments, and the presence of dormant persister cells [12].

In body sites naturally colonized by commensal microbiota, such as the gastrointestinal and female reproductive tracts, the presence of biofilms does not necessarily represent a pathological condition and may instead constitute part of the normal physiological microbial environment [13]. Moreover, these commensal biofilm-associated microbial communities contribute significantly to colonization resistance by limiting the establishment and proliferation of invading pathogenic microorganisms, thereby helping to maintain tissue homeostasis and protect against the development of infection [14].

In contrast, bacterial biofilm formation in normally sterile body sites, or disturbances affecting naturally colonized microbial communities, may disrupt the natural balance of the microbiota and contribute to the development of pathological conditions associated with infectious disease. Clinically, biofilms are frequently identified on mucosal surfaces, within the oral cavity, and on indwelling medical devices such as catheters [15,16], prosthetic joints [17], and cardiac implants [18]. In particular, infections associated with indwelling medical devices represent a major clinical challenge, as these devices are indispensable in modern surgical and medical practice, while their treatment often requires complex therapeutic interventions that further increase the risk of complications and adverse patient outcomes [19]. According to the conclusions of the NIH (National Institutes of Health), microbial biofilm formation is involved in 65% of all infections and 80% of chronic infections [20].

The most frequently identified etiological agents associated with infections on indwelling medical devices are coagulase-negative staphylococci, predominantly *Staphylococcus epidermidis*, and *Staphylococcus aureus* [21,22]. Multidrug-resistant Gram-negative bacteria, including *Escherichia coli*, *Klebsiella pneumoniae*, *Pseudomonas aeruginosa*, and *Acinetobacter baumannii*, are also capable of inducing medical device-associated infections, particularly within complex hospital environments [23].

### 2.1. Biofilm Formation

Biofilm formation is typically characterized as a three-stage process: (i) initial attachment of microorganisms to a surface, (ii) subsequent proliferation and development of a mature, structurally organized biofilm, and (iii) detachment, commonly referred to as dispersion [24].

The extent of bacterial adhesion to abiotic surfaces is governed by physico-chemical properties, among which surface hydrophobicity represents a principal determining factor. However, adhesion to abiotic surfaces is likely to play only a minor role in biofilm formation under in vivo conditions. In the human body, bacterial attachment to tissues or implanted medical devices is predominantly mediated by interactions with host extracellular matrix proteins, which rapidly adsorb onto device surfaces following implantation [25]. For instance, the major underlying molecules for *Staphylococcus aureus* are a class of surface-attached bacterial proteins known as MSCRAMMs (microbial surface components recognizing adhesive matrix molecules) [26]. During the initial phase of biofilm development, microorganisms establish a loose and reversible association with the surface. Subsequently, a number of reversibly adsorbed cells remain immobilized, leading to irreversible adhesion and the establishment of resistance to various physical factors that would otherwise impede biofilm formation [27].

Following successful adhesion to the surface, the attached microorganisms undergo active proliferation and aggregation within a self-produced extracellular polymeric substance (EPS) matrix, culminating in the formation of microcolonies [28]. The extracellular polymeric substance (EPS) is fundamental to biofilm maturation, stabilizing the three-dimensional biofilm architecture, facilitating microbial adhesion to surfaces, and promoting cellular aggregation. Additionally, EPS confers protection against diverse stressors, including host immune responses, antimicrobial agents, oxidative damage, and metal ions, while also encapsulating signaling molecules essential for quorum sensing, as well as metabolic byproducts and enzymes [29]. A mature biofilm exhibits a three-tiered organization: high-density layer, low-density layer and microenvironmental layer [30].

The last stage in the developmental life cycle of the biofilm is referred to as dispersion. In general, mechanisms of biofilm dispersion can be divided into active and passive. Active dispersion is mediated by a reduction in intracellular cyclic diguanylate monophosphate (c-di-GMP) levels, which induces the production of enzymes responsible for degrading the biofilm matrix and facilitating cellular dispersion. Passive dispersion is driven by external triggers that directly promote the detachment of cells from the biofilm [31].

### 2.2. Current Treatment Options and Their Limitations

Biofilms are capable of inducing disease within the host through both device-associated and non–device-associated infections. According to estimates by the National Institutes of Health (NIH), biofilms are implicated in up to 80% of all bacterial infections in humans [32].

In antibiotic therapy, it is crucial to administer a dosage sufficiently high to achieve effective drug concentrations at the site of infection, while remaining within the safety thresholds for renal and hepatic function. Combination therapy is frequently favored in place of monotherapy. Continuous surveillance of infection progression enables timely optimization of the antibiotic regimen, encompassing modifications in dosage, treatment duration, or antimicrobial selection, according to the patient’s clinical response and the specific characteristics of the biofilm [32]. However, prolonged antibiotic therapy has been reported to be only sometimes adequate in the management of non–device-associated infections [33].

It is strongly recommended that infected indwelling medical devices be removed or, when clinically indicated, replaced with sterile substitutes. The presence of a foreign body inherently provides an optimal surface for bacterial adhesion and subsequent biofilm development. Only partial reduction in the biofilm can typically be achieved, often resulting in relapse accompanied by exacerbation of the infection [33]. Furthermore, the presence of a foreign body has been reported to significantly suppress both the phagocytic activity and the intracellular bactericidal functions of polymorphonuclear leukocytes [34].

Building on the limitations of both antibiotic therapy and surgical removal, emerging scientific perspectives are shifting toward innovative approaches that disrupt bacterial communication and regulations at the molecular level. Such as quorum-sensing inhibitors, anti-quorum-sensing peptides, modification of c-di-GMP and disruption of bacterial amyloids [33].

## 3. Micro/Nanorobots as a Potential Therapeutic Tool

The origins of nanoscience and nanotechnology are commonly traced to Richard Feynman’s seminal 1959 lecture to the American Physical Society, entitled “There’s Plenty of Room at the Bottom.” [35]. Public fascination with this previously unseen domain and its transformative potential is reflected in popular culture, for example, in the classic 1966 science-fiction film Fantastic Voyage, in which a submarine and its crew are miniaturized to the microscale to navigate the human vasculature in order to remove a blood clot. To investigate the feasibility of this once-speculative concept, researchers have proposed and developed a wide range of miniature machines and robotic systems, several of which are designed to function within diverse physiological environments for diagnostic and therapeutic applications. The term medical micro/nanorobots is used to denote nano- to microscale structures (approximately 300 nm to 300 µm) that are capable of transducing various power sources into kinetic energy [36]. As small-scale entities, micro/nanorobots possess the capacity to perform functional tasks with partial or complete autonomy within otherwise inaccessible regions of the body. In this context, their ability to actively locomote constitutes a substantial advantage [37,38]. Three principal categories of powered micro/nanorobots are typically distinguished: (i) biohybrid systems, which couple synthetic nanostructures with motile microorganisms that serve as the propulsion mechanism; (ii) chemically powered micro/nanorobots, which employ asymmetric catalytic architectures to convert specific chemical fuels into directed motion; and (iii) physically powered micro/nanorobots, which transduce externally applied energy inputs, such as magnetic, acoustic, or optical fields, into translational movement through engineered geometries and material properties [36]. Beyond their locomotion capabilities, micro/nanorobots offer extensive opportunities for structural and functional customization—including cargo loading, surface coating, and functionalization—tailored to the requirements of specific applications [39]. These intentionally engineered modifications enable them to benefit from stimuli-responsive properties [9] and empower them with intrinsic controllability [8], which could be leveraged to minimize side effects while improving treatment efficacy [10]. At present, the application of nanorobots is extensively investigated under in vitro conditions; however, experimental in vivo studies, which constitute the focus of this review, remain comparatively scarce.

### 3.1. Antimicrobial Mechanisms and Strategies of Micro/Nanorobots

**Figure 1:**
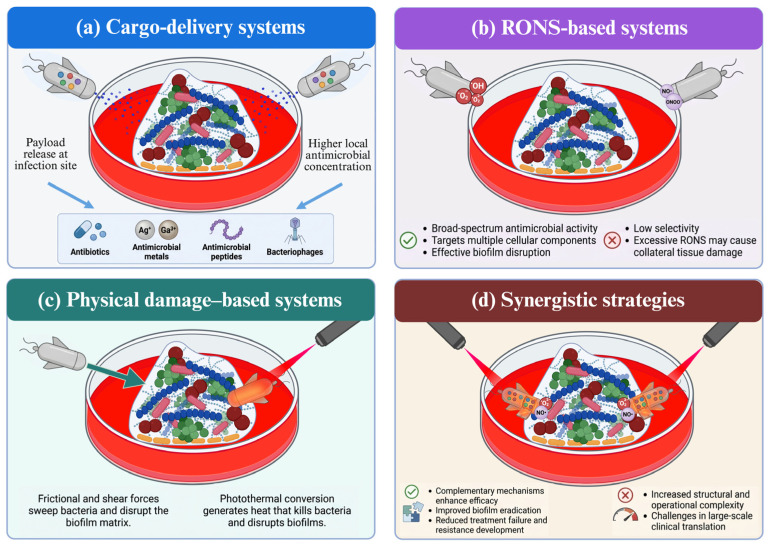
Schematic overview of antimicrobial strategies employed by micro/nanorobots: (**a**) cargo-delivery systems, highlighting site-specific release of antimicrobial agents and increased local therapeutic concentration; (**b**) reactive oxygen and nitrogen species (RONS)-based systems, emphasizing their principal advantages and limitations; (**c**) physical damage-based systems, illustrating their classification into mechanical and non-mechanical antimicrobial mechanisms; and (**d**) synergistic strategies, highlighting their combined therapeutic benefits and associated limitations. Created in BioRender. Musil, O. (2026) https://BioRender.com/xm48fnz (accessed on 30 April 2026).

In contrast to passive diffusion-based systems, dynamic micro/nanorobots enable targeted therapeutic action. Furthermore, their intelligent responsiveness allows real-time sensing of the infectious microenvironment and facilitates access to otherwise difficult-to-reach regions of the human body through either autonomous or externally guided actuation. The strategies of active bactericidal action employed by micro/nanorobots are generally categorized into four classes: (a) Cargo-delivery systems, which lower the required therapeutic dose, increase local drug concentration, and decrease the likelihood of resistance development; (b) Reactive oxygen/nitrogen species based systems, which disrupt bacterial membranes and exhibit potent, broad-spectrum bactericidal activity; (c) Physical damage-based mechanisms, which can be remotely actuated and do not induce antimicrobial resistance; and (d) Synergistic strategies, which integrate the aforementioned mechanisms [40,41] (Figure 1, Table 1).

#### 3.1.1. Antibacterial Therapeutic Cargo Delivery Approaches

In antimicrobial cargo-delivery approaches, micro/nanorobots function as actively propelled or externally guided carriers that enhance targeted drug transport with high precision, improve penetration into biofilm matrices, and prolong retention at infection sites compared with passive delivery systems [42]. Antibiotics continue to play a central role in conventional antimicrobial therapy. Their mechanisms of action include disruption of bacterial cell membranes, inhibition of deoxyribonucleic acid (DNA) replication and repair, suppression of protein synthesis, and interference with essential metabolic processes [43]. However, the microorganisms targeted by these agents progressively develop resistance, a process that is further accelerated by the widespread overuse of antibiotics in clinical practice. The use of micro/nanorobots as carriers for antibiotic delivery can increase the local concentration of active antibiotics, reduce the required therapeutic dosage, and lower the likelihood of resistance development. In 2017, de Ávila et al. demonstrated a magnesium-based micromotor comprising a clarithromycin-loaded poly(lactic-co-glycolic acid) (PLGA) layer and an outer chitosan polymer coating surrounding a Mg propellant core and a titanium dioxide (TiO_2_) shell for the in vivo treatment of *Helicobacter pylori* infection in a murine model [44]. Benefiting from the intrinsic properties of magnesium-based micromotors, which utilize gastric acid as fuel, thereby enabling autonomous self-propulsion and active distribution throughout the gastric environment [45], as well as from adhesion to the gastric mucosa mediated by the positively charged chitosan outer coating [46], this system enables efficient, localized, and autonomous release of clarithromycin from the PLGA polymer layer during motor dissolution. Two weeks after inoculation with *Helicobacter pylori*, mice were orally administered clarithromycin-loaded magnesium-based micromotors once daily for five consecutive days, resulting in an approximately 1.8 orders of magnitude reduction in bacterial burden compared with the negative control group. In 2021, Shchelik et al. developed biohybrid microswimmers exhibiting intrinsic phototactic behavior, which enabled directional navigation and spatially controlled antibiotic delivery by covalently conjugating vancomycin and ciprofloxacin to the surface of *Chlamydomonas reinhardtii* via a photocleavable linker [47]. This strategy enabled three-dimensional spatial localization of antibiotic activity within the region of interest and on-demand drug release upon UV irradiation (λ = 365 nm) in vitro, resulting in a three orders of magnitude reduction in the growth of methicillin-resistant *Staphylococcus aureus* (MRSA) and *Escherichia coli* compared with control groups. In 2022, Zhang et al. developed hybrid microrobots for the active delivery of ciprofloxacin to the lungs of a murine model in vivo by attaching antibiotic-loaded neutrophil membrane-coated poly(lactic-co-glycolic acid) (PLGA) nanoparticles to natural microalgae *Chlamydomonas reinhardtii* (denoted ‘algae-NP(Cip)-robot’), thereby combining the autonomous motility of living microalgae with targeted nanoparticle-mediated antibiotic delivery in order to treat pneumonia caused by *Pseudomonas aeruginosa* [48]. The distribution of the hybrid microrobots lacking the antibiotic payload (denoted ‘algae-NP-robot’) was assessed by ex vivo fluorescence imaging of excised lungs at multiple time points. These results were compared with those of a deflagellated, static control group (denoted ‘static algae-NP’), demonstrating significantly enhanced lung retention of the ‘algae-NP-robot’ and illustrating the contribution of active microswimmer propulsion for pulmonary retention. Following intratracheal inoculation of *Pseudomonas aeruginosa*, treatment with algae-NP(Cip)-robot samples was intratracheally administered, which resulted in a three orders of magnitude reduction in bacterial colony-forming units compared with the negative control, and a significant reduction compared with static algae-NP(Cip). Furthermore, a survival study was conducted under an identical experimental protocol, in which the group of mice treated with algae-NP(Cip)-robot samples exhibited 100% survival throughout the 14-day study period, in contrast to the untreated group, in which all mice died within three days.

Alternatively, silver and other metal-based nanoparticles exhibit broad-spectrum antibacterial activity and a reduced propensity to induce microbial resistance, owing to their ability to act on multiple cellular targets [40,41,49]. In 2016, Hoop et al. proposed a strategy for targeting and eliminating *Escherichia coli* in vitro using silver-coated magnetic nanocoils capable of magnetic field-guided locomotion and active interaction with bacterial cells [50]. To impart both magnetic and antibacterial properties, the outer surface of the Pd nanocoils was coated with a uniform thin layer of Ni and Ag. The dynamic bactericidal activity of the Pd/Ni/Ag nanocoils was assessed by colony-forming unit (CFU) counting over a concentration range of 5–100 ppm at multiple time points. Treatment with 100 ppm resulted in a six-order-of-magnitude reduction in bacterial viability within 2 h, with the minimum bactericidal concentration of the Ag-coated nanocoils determined to be 30 ppm. At lower concentrations (5 and 10 ppm), the nanocoils were still able to inhibit bacterial growth after 2 h of incubation relative to the untreated control. In 2021, Lin et al. reported a hydrogen-bubble-propelled Janus gallium/zinc (Ga/Zn) micromotor for the active in vitro treatment of Helicobacter pylori [51]. Zinc microparticles were employed as templates and subsequently coated asymmetrically with gallium. The presence of gallium galvanically enhanced the Zn–acid reaction, facilitating propulsion via hydrogen bubble generation and simultaneously releasing Ga^3+^ ions, which exhibit enhanced antibacterial activity. Subsequently, the Ga/Zn microrobots achieved 99.9% antibacterial efficacy at only one-fourth of the minimum inhibitory concentration (MIC) required for free gallium.

Furthermore, antimicrobial peptides (AMPs) have demonstrated robust antimicrobial activity against drug-resistant bacterial infections [52,53]. AMPs employ an array of distinct mechanisms to neutralize bacteria while maintaining low toxicity toward normal cells and tissues [54], in contrast to the high toxicity and pronounced oxidative stress often associated with nanometal-coated micro/nanorobots. In 2020, Yuan et al. reported a selective capture/kill microrobot that integrates nisin with GO/PtNPs/Fe_2_O_3_ catalytic and/or magnetic Janus micromotors whose self-propelled and magnetically controllable motion enhanced contact between AMP and its prey for in vitro targeting of *Staphylococcus aureus* [55]. Nisin is a lantibiotic that exhibits specific antimicrobial activity against Gram-positive bacteria by binding to lipid II in the bacterial membrane, thereby disrupting membrane integrity and inducing leakage of intracellular contents [56]. By leveraging this micromotor-based strategy, a twofold enhancement in capture and killing efficiency was achieved compared with the free peptide and static control counterparts. Additionally, lysozyme, a naturally occurring antimicrobial enzyme abundantly present in bodily secretions, including tears and saliva, has been demonstrated to exhibit antimicrobial activity and a well-established role in host defense against microbial pathogens [57]. Through hydrolysis of the β-1,4-glycosidic bond between N-acetylglucosamine and N-acetylmuramic acid within the peptidoglycan layer of the bacterial cell wall, lysozyme disrupts cell wall integrity, ultimately leading to bacterial cell lysis and death [58]. Enzymes such as lysozymes are typically optimized to function under relatively stable physiological conditions; consequently, their activity and stability may be compromised under the harsh and variable environments associated with in vivo infections. However, integration with nanoparticles has the potential to mitigate these limitations and enhance enzymatic stability and therapeutic efficacy [59]. In 2015, Kiristi et al. designed ultrasound-propelled porous gold nanowire motors in which acoustic propulsion increased collision frequency between nanomotors functionalized with lysozyme and bacterial cells and demonstrated their effectiveness against Gram-positive *Micrococcus lysodeikticus* and Gram-negative *Escherichia coli* in vitro [60]. By coupling the intrinsic antibacterial activity of the enzyme with the rapid propulsion of the porous gold nanowires, this system enhances enzyme–bacterium interactions and prevents surface aggregation of dead bacterial cells, thereby resulting in a markedly improved bactericidal efficacy. The observed differences in killing efficiencies against *M. lysodeikticus* (84%) and *E. coli* (70%) further demonstrate the higher antimicrobial efficacy of lysozyme toward the former organism, attributable to structural differences in the cell walls of Gram-positive and Gram-negative bacteria.

Alternatively, bacteriophage-coated micro/nanorobots have been reported as promising targeted antimicrobial platforms. These systems employ engineered bacteriophages capable of delivering CRISPR-associated proteins into bacterial cells, where they cleave and disrupt bacterial DNA, leading to cell death [61]. In 2017, Li et al. demonstrated in vitro the use of Fe_3_O_4_-based, chitosan-coated magnetic colloidal nanoparticle clusters functionalized with polyvalent bacteriophages (PEL1) via amide linkages for the removal of biofilms formed by *Pseudomonas aeruginosa* and *Escherichia coli* [62]. A biofilm removal efficiency of 88.7 ± 2.8% was achieved, attributable to elevated local phage concentrations and enhanced penetration, compared with 35.5 ± 6.6% observed in the treatment with free bacteriophages. In 2019, Yu et al. investigated in vitro the impact of chitosan-coated Fe_3_O_4_ magnetic colloidal nanoparticle clusters of varying sizes, covalently conjugated with polyvalent bacteriophages PEB1 or PEB2, on dual and multi-species biofilm removal efficiency [63]. These clusters were capable of externally guided transport and enhanced penetration into biofilm structures under magnetic actuation. Smaller conjugates were observed to distribute phages more uniformly throughout the basal layer of the biofilm, resulting in enhanced disruption of this layer and achieving removal efficiencies of 98.3 ± 1.4% for dual-species biofilms and 92.2 ± 3.1% for multispecies biofilms. In contrast, larger conjugates promoted vertical propagation within the biofilm but exhibited lower removal efficiencies of 80.2 ± 3.4% for dual-species biofilms and 67.6 ± 3.8% for multispecies biofilms due to reduced horizontal diffusion at the biofilm base.

#### 3.1.2. Antibacterial Strategies Based on the Generation of Reactive Oxygen and Nitrogen Species

In these approaches, micro/nanorobots enhance the bactericidal efficacy of reactive oxygen and nitrogen species through active transport, improved biofilm penetration, and localized generation of reactive intermediates at the infection site [41]. Reactive oxygen and nitrogen species (RONS) comprise highly reactive and unstable molecules, ions, and radicals, including reactive oxygen species (ROS) and reactive nitrogen species (RNS). These species induce tissue damage through oxidative stress, resulting in the degradation of essential cellular components such as lipids, proteins, and DNA [64]. The ensuing generation of secondary reactive species can trigger diverse cellular responses and ultimately lead to cell death via apoptotic or necrotic pathways [65]. The term “oxidative stress” was originally defined by Sies in 1985 as an imbalance between oxidants and antioxidants in favor of oxidants [66]. However, this concept has since evolved to encompass signaling processes as well, giving rise to the field of redox biology [67]. Owing to these aforementioned capabilities, as well as their ability to degrade the extracellular polymeric substances (EPS) of biofilms, mobile RONS-generating platforms have attracted substantial scientific interest [40]. In 2019, Xu et al. fabricated a urea-driven hollow mesoporous SiO_2_ micromotor loaded with the photosensitizer 5,10,15,20-tetrakis(4-aminophenyl)porphyrin, which effectively eradicated *Escherichia coli* in vitro [68]. The singlet oxygen generation efficiency of the mobile photosensitizer was enhanced by more than 20%, and its active propulsion increased the effective treatment area by approximately tenfold. These enhancements were confirmed by flow cytometry analysis and fluorescence microscopy observations, in comparison with passive carrier systems under identical conditions. In 2018, Yu et al. developed a polydopamine-coated iron oxide nanocomposite grafted with dendritic poly(amidoamine) and loaded with nitric oxide (NO) to enable reactive nitrogen species (RNS) release under intermittent 808 nm laser irradiation, resulting in effective in vitro eradication of *Escherichia coli* and *Staphylococcus aureus* [69]. In 2021, Lu et al. proposed a magnetically guided nanoworm composed of citrate-capped Au–Ag nanoparticles loaded with Fe_2_O_3_ core and coated with an L-arginine–modified polydopamine (PDA) outer shell enabling externally controlled accumulation at the infected site for in vivo targeted, near-infrared–triggered bacterial eradication [70]. Using a male BALB/c mouse model, *Staphylococcus aureus* was injected into the right leg muscle to establish a localized abscess over a two-day period. Subsequently, fluorescent rhodamine B (Rho-B)–labeled nanoworms were administered intravenously. Following 30 min of magnetic field exposure at the infected site, nanoworm accumulation was approximately 4.2-fold higher than that observed in the control group, demonstrating their targeted delivery capability. Furthermore, in vivo inhibition of abscess growth was demonstrated both clinically and histopathologically after 15 days of treatment activated by near-infrared (NIR) laser irradiation, which was attributed to nitric oxide (NO) generation, as established in a previous in vitro experiment.

#### 3.1.3. Physical Damage Mediated by Micro/Nanorobots

Due to their nanoscale dimensions, micro/nanorobots are capable of directly interacting with cells and, in some cases, penetrating cellular membranes [71]. Through mechanically or non-mechanically mediated external interventions, they can induce membrane disruption or intracellular damage, ultimately leading to cell death. Furthermore, mechanical intervention leverages the ability of micro/nanorobots to generate frictional and shear forces, thereby directly disrupting and degrading the extracellular matrix of biofilms [40]. In 2020, Elbourne et al. reported the use of magneto-responsive, gallium-based Fe-loaded liquid metal droplets for the in vitro disruption and eradication of bacterial biofilms [72]. The application of a low-intensity rotating magnetic field induced coordinated actuation and mechanical deformation of the liquid metal, forming sharp-edged structures capable of physically rupturing bacterial cells and disrupting the dense biofilm matrix. After 90 min of magnetic field exposure, more than 99% of both *Pseudomonas aeruginosa* and *Staphylococcus aureus* cells were rendered nonviable, and the biofilm structures were substantially disintegrated.

Alternatively, non-mechanical cell lysis relies on the delivery of externally applied energy to induce bacterial inactivation, including thermal, photonic, electrical, pressure-based, and acoustic modalities. In particular, photothermal therapy (PTT) has recently attracted considerable research interest due to its advantages, including non-invasiveness, low toxicity, operational simplicity, broad-spectrum antibacterial efficacy, and a reduced likelihood of inducing drug resistance [73]. PTT refers to a therapeutic strategy in which a photothermal agent (PTA) converts light into heat upon irradiation, thereby inducing irreversible bacterial damage through thermal effects, including protein denaturation and disruption of membrane integrity [74]. In 2023, Wang et al. engineered a biomimetic aggregation-induced emission (AIE) nanorobot functionalized with neutrophil membranes on its surface (termed CM@AIE NPs), consisting of NIR-II-responsive AIE photothermal nanoparticle cores camouflaged with neutrophil cell membranes [75]. This construct was capable of precise targeting through interactions between neutrophil membrane components and inflammatory signaling molecules, as well as facilitating photothermal effects on inflammatory sites under NIR-II laser irradiation. The in vitro antibacterial efficacy was evaluated against *Staphylococcus aureus* and *Escherichia coli*, demonstrating that treatment with CM@AIE NPs combined with NIR-II laser irradiation reduced the bacterial survival rates to 6.3% and 5.6%, respectively. Additionally, this novel nanorobot is anticipated to exhibit precise targeting capabilities by engaging with immunomodulatory molecules that typically recognize neutrophils, resulting in substantial accumulation at inflammatory wound sites in mice 36 h following intravenous administration. Thermal imaging revealed that the MRSA (methicillin-resistant *Staphylococcus aureus*)-infected wound site underwent rapid heating upon exposure to a 980 nm laser, whereas only a slight temperature increase was observed in the control groups, indicating that CM@AIE NPs induced localized hyperthermia. On day 14, wounded skin samples were collected for H&E and Masson’s trichrome analyses, which demonstrated enhanced wound healing in mice treated with CM@AIE NPs combined with NIR-II laser irradiation compared to the control groups.

However, hyperthermia can also be exploited as an effective antimicrobial strategy through approaches other than photothermal therapy, such as nanoparticle-mediated magnetic hyperthermia. In this method, electromagnetic energy is converted into heat when subjected to an external high-frequency alternating magnetic field [76]. In 2016, Chen et al. demonstrated this phenomenon by coating magnetotactic MO-1 bacteria with anti-MO-1 polyclonal antibodies, which facilitated the attachment of MO-1 cells to *Staphylococcus aureus* [77]. Upon exposure to an alternating magnetic field, the resulting hyperthermia achieved a bacterial killing efficiency exceeding 50% in vitro. Furthermore, in vivo studies using mouse models revealed significantly enhanced wound healing.

#### 3.1.4. Synergistic Combinatorial Approaches

Synergistic approaches have been shown to effectively integrate the complementary advantages of antimicrobial cargo delivery systems, reactive oxygen and nitrogen species (RONS) based modalities, and mechanisms of physical disruption. The conventional integration of physical therapeutic modalities with traditional antimicrobial agents has been shown to enhance bactericidal efficacy, particularly against antibiotic-resistant pathogens, while also reducing the likelihood of infection recurrence [40]. In 2020, Bhuyan et al. reported the development of biocompatible micromotors derived from tea buds, termed T-Budbots, which are capable of magnetic propulsion across biofilm matrices [78]. These micromotors were functionalized with ciprofloxacin via electrostatic interactions at their surface, thereby enabling the implementation of a “Kill-n-Clean” strategy against *Pseudomonas aeruginosa* and *Staphylococcus aureus* in vitro. By leveraging both their active propulsion to efficiently eradicate and mechanically disrupt established biofilms, as well as the pH-responsive release of ciprofloxacin within the acidic biofilm microenvironment, effective biofilm dismantling was achieved. Additionally, as a further example of the synergistic integration of cargo delivery and physical disruption, distinct from conventional antibiotic loading, the combined effects of metal-based nanoparticles and mechanical capture have been reported. In 2017, Vilela et al. reported the development of self-propelled Janus micromotors constructed from a magnesium microparticle core that served both as a structural template and as a propulsion source through hydrogen bubble generation upon contact with water [79]. The micromotors incorporated an intermediate iron layer to enable magnetic guidance and retrieval, as well as an outer gold layer decorated with silver nanoparticles (AgNPs) to facilitate bacterial adhesion and enhance bactericidal activity, thereby enabling the capture and subsequent eradication of *Escherichia coli*. By leveraging the combination of the aforementioned strategies, AgNP-coated Janus micromotors achieved efficient bactericidal performance, eliminating more than 80% of *Escherichia coli* within 15 min in contaminated aqueous solutions. In contrast, colloidal silver nanoparticles under comparable conditions resulted in less than 35% bacterial inactivation.

Alternatively, the synergistic coupling of physical disruption with reactive oxygen species (ROS) generation has demonstrated considerable potential for enhancing the bactericidal efficacy of free radicals, particularly in the context of complex biofilm dismantling. In 2021, Dong et al. developed magnetic porous Fe_3_O_4_ mesoparticles (p-Fe_3_O_4_ MPs) designed to exploit the synergistic effects of chemical and physical processes, including the generation of bactericidal free radicals via the Fenton reaction, mechanical disruption of biofilms, and enhanced penetration of reactive oxygen species (ROS) into biofilm matrices through collective swarm motion [80]. The antibacterial efficacy of these mesoparticles was evaluated against *Escherichia coli* and *Bacillus cereus* biofilms, achieving killing efficiencies of 99.99% and 98%, respectively.

#### 3.1.5. Discussion

Following the presented studies, it becomes evident that micro/nanorobotic systems represent highly versatile antimicrobial platforms capable of integrating targeted delivery, active propulsion, environmental responsiveness, and externally controlled actuation into a single therapeutic strategy. Although the individual antimicrobial approaches differ substantially in their mechanisms of action, all of them aim to overcome the principal limitations associated with conventional antimicrobial therapies.

Cargo-delivery approaches primarily benefit from enhanced localization and prolonged retention of antimicrobial agents at infection sites, thereby improving therapeutic efficacy while reducing systemic exposure and required dosages. In comparison with passive delivery systems, micro/nanorobots can actively penetrate dense extracellular polymeric substance (EPS) matrices and achieve site-specific payload release, resulting in higher local antimicrobial concentrations and improved biofilm disruption. However, the therapeutic efficacy of these systems remains dependent on the selected cargo. Conventional antibiotics still face the fundamental limitation of pre-existing antimicrobial resistance. In contrast, antimicrobial metals exhibit broad-spectrum and multi-target activity that reduces the likelihood of resistance development, although this advantage is counterbalanced by limited selectivity, cytotoxicity, and excessive oxidative stress. Comparatively, antimicrobial peptides generally demonstrate lower cytotoxicity toward host tissues and broad-spectrum antibacterial activity, but their stability and activity may be compromised under the harsh conditions associated with in vivo infections. In this regard, nanorobotic carriers may improve their stability and retention at infection sites. Bacteriophages represent another promising cargo, particularly for the treatment of antibiotic-resistant bacteria. However, their narrow pathogen identification may limit broader clinical applicability.

RONS-generating systems provide efficient broad-spectrum antimicrobial activity through localized oxidative stress, enabling both bacterial destruction and degradation of the extracellular polymeric substance (EPS) matrix. Similar to antimicrobial metals, these systems are less susceptible to conventional resistance mechanisms because they target multiple cellular structures simultaneously. However, their low selectivity remains a major limitation, as excessive reactive species production may also induce collateral tissue toxicity and damage.

Physical damage-based systems offer an alternative strategy. A major advantage of these approaches is that they do not rely primarily on biochemical antimicrobial mechanisms and are therefore less likely to contribute to antimicrobial resistance development. Furthermore, their ability to physically disrupt the extracellular polymeric substance (EPS) matrix directly addresses one of the principal barriers to effective biofilm eradication. Nevertheless, non-mechanical approaches may still cause collateral host tissue damage due to excessive localized heat generation.

Synergistic systems appear particularly promising because they integrate complementary antimicrobial mechanisms, thereby enhancing therapeutic efficacy while potentially reducing treatment failure and resistance development. However, this improved functionality is accompanied by substantially increased structural and operational complexity, which may hinder large-scale clinical translation.

Despite these promising findings, direct comparison between individual micro/nanorobotic platforms remains challenging due to substantial variability in experimental methodologies and evaluation criteria across studies. The currently available literature employs a broad range of antibacterial and antibiofilm outcome measures, including colony-forming unit (CFU) reduction, fluorescence-based live/dead staining, flow cytometry, MIC/MBC-related antibacterial evaluation, optical density measurement, CLSM, SEM, TEM, FETEM and FESEM imaging, histopathological assessment, wound healing analysis, and survival studies in animal models (Table 2). Furthermore, differences in bacterial strains, biofilm maturation stages, propulsion mechanisms, treatment durations, and in vitro versus in vivo experimental conditions further complicate interstudy comparisons and translational interpretation of therapeutic efficacy.

**Table 1:** Antimicrobial mechanisms and strategies of micro/nanorobots, including their associated advantages and constraints.

Strategy		Mechanism	Benefit	Drawback	Ref.
Cargo delivery	Antibiotics	Selective toxicity by targeting essential bacterial structures or processes	Site-specific drug release improving therapeutic efficacy and reducing systemic adverse effects	Presence of existing antibiotic resistance	[44,47,48]
	Antimicrobial metals	Microbial cell death resulting from membrane disruption, protein inactivation, DNA damage, and metabolic interference	A multi-target mechanism that limits the ability of microorganisms to develop resistance	Broad cytotoxicity	[50,51]
	Antimicrobial peptides	Interaction with negatively charged membranes, disruption of membrane, and, in some instances, interference with intracellular targets	Low cytotoxicity to normal cells and tissues and broad spectrum	May contribute to the development of antimicrobial resistance	[55,60]
	Bacteriophages	Intracellular takeover and cell rupture	Elimination of antibiotic-resistant bacteria	Highly specific target recognition	[62,63]
Reactive oxygen and nitrogen species generation	Reactive oxygen species	Damage to bacterial membranes and DNA, along with interference in metabolic processes	Degradation of the extracellular polymeric substances (EPS) of biofilms	Low selectivity leading to tissue toxicity	[68]
	Reactive nitrogen species				[69,70]
Physical damage	Mechanical	Frictional and shear forces	Does not contribute to the development of antimicrobial resistance	May damage host tissue	[72]
	Non-mechanical	Externally applied energy shock			[75,77]
Synergistic strategies		Synergistic combination of the previously discussed	Enhanced bactericidal efficacy and biofilm dismantling	Increased complexity	[78,79,80]

**Table 2:** Summary of the presented studies illustrating specific antimicrobial strategies and corresponding outcome measurements.

Study	Strategy	Antibacterial Outcome Measurement	Ref.
de Ávila et al. (2017)	Cargo delivery of clarithromycin	CFU quantification	[44]
Shchelik et al. (2021)	Cargo delivery of vancomycin and ciprofloxacin	CFU quantification	[47]
Zhang et al. (2022)	Cargo delivery of ciprofloxacin	CFU quantification and survival analysis	[48]
Hoop et al. (2016)	Silver ions mediated antibacterial delivery	CFU quantification, LIVE/DEAD bacterial viability assay, and SEM imaging	[50]
Lin et al. (2021)	Gallium ions mediated antibacterial delivery	MIC/MBC-related antibacterial evaluation and optical density measurement	[51]
Yuan et al. (2021)	Nisin mediated antibacterial delivery	LIVE/DEAD viability assay, fluorescence microscopy-based capture/killing and biofilm disruption analyses, and SEM imaging	[55]
Kiristi et al. (2015)	Lysozyme mediated antibacterial delivery	Spectrophotometric optical density, fluorescence, and SEM viability imaging	[60]
Li et al. (2017)	Bacteriophages mediated antibacterial delivery	LIVE/DEAD viability assay	[62]
Yu et al. (2019)	Bacteriophages mediated antibacterial delivery	CFU quantification, LIVE/DEAD viability assay, and optical density measurement	[63]
Xu et al. (2019)	Reactive oxygen species generation	LIVE/DEAD bacterial viability assay, flow cytometry	[68]
Yu et al. (2018)	Reactive nitrogen species generation	CFU quantification of surviving, LIVE/DEAD bacterial viability assay, optical density measurement, and SEM imaging	[69]
Lu et al. (2021)	Reactive nitrogen species generation	CFU quantification, LIVE/DEAD viability assay, optical density measurement, SEM and CLSM imaging, histopathological analysis, and in vivo abscess assessment	[70]
Elbourne et al. (2020)	Mechanical physical damage	CFU quantification, optical density measurement, SEM, TEM and CLSM imaging	[72]
Wang et al. (2023)	Non-mechanical physical damage	CFU quantification, LIVE/DEAD viability assay SEM, TEM and thermal imaging, histopathological analysis, and in vivo abscess assessment	[75]
Chen et al. (2016)	Non-mechanical physical damage	Flow cytometry, in vivo infected wounds lengths measurements	[77]
Bhuyan et al. (2020)	Synergistic combination of mechanical physical damage and ciprofloxacin cargo delivery	LIVE/DEAD viability assay, optical density measurement, FETEM and FESEM imaging	[78]
Vilela et al. (2017)	Synergistic combination of mechanical physical capture and silver ions mediated antibacterial delivery	LIVE/DEAD viability assay and SEM imaging	[79]
Dong et al. (2021)	Synergistic combination of mechanical physical damage and reactive oxygen species generation	CFU quantification, LIVE/DEAD viability assay, optical density measurement TEM images	[80]

## 4. In Vivo Applications of Micro/Nanorobots for Biofilm Eradication

As discussed in greater detail in the section on biofilms in the human body, biofilm-associated infectious diseases exert a substantial impact on human health [20,32]. These infections encompass a broad spectrum of pathological conditions, including indwelling device-associated infections [15,16,17,18,81,82,83], dental caries [84], osteomyelitis [85], otitis media [86], sinusitis [87], and cystic fibrosis-associated pulmonary infections [88]. In addition to bacterial cells, biofilms are predominantly composed of extracellular polymeric substances (EPS) secreted by microorganisms, including polysaccharides, proteins, lipids, and nucleic acids [89]. This extracellular matrix confers protective functions, effectively shielding embedded bacteria from diverse antimicrobial agents and mechanisms [12], such as antibiotics, metal ions, oxidizing species, antimicrobial peptides (AMPs), and host immune responses. The increasing prevalence of antibiotic resistance, together with the consequent decline in the efficacy of conventional therapeutic strategies, is driving the exploration and development of novel approaches for the treatment of biofilm-associated infections [33] (Table 3).

### 4.1. Eradication of Indwelling Device-Associated Biofilm Infections In Vivo

The use of indwelling medical devices, including urinary catheters, central venous and peripherally inserted central catheters, as well as endotracheal tubes, has become an integral component of patient management in hospitalized settings, particularly in intensive care practice. Similarly, osteosynthetic plates, prosthetic joints, shunts, and cardiac valves are widely employed in diverse surgical applications and are frequently retained in situ for extended periods. Among the most significant causes of device failure are microbial infections and subsequent biofilm formation, which are associated with increased morbidity and mortality rates and contribute substantially to escalating healthcare costs [90]. Furthermore, the conventional management of indwelling device-associated infections remains highly challenging and frequently necessitates surgical removal of the implanted device. This clinical limitation has emerged as a significant driving force for ongoing research aimed at developing more effective therapeutic strategies. In 2020, Yu et al. engineered a multifunctional nanosystem composed of large-pore mesoporous silica nanoparticles (MSNLPs) capped with β-cyclodextrin (β-CD)–modified polyethylenimine (PEI-CD), together with adamantane (ADA)-functionalized MSNs incorporating a magnetic core (MagNP@MSNA) and capped with cucurbit[6]uril (CB[6]) co-assemblies [91]. This platform was designed to exploit the synergistic antibacterial effects of the antimicrobial peptide melittin (MEL) and the low-molecular-weight antibiotic ofloxacin (OFL). The therapeutic efficacy of the system was evaluated for the eradication of *Pseudomonas aeruginosa* biofilms formed on silicone tubes implanted in six-week-old male BALB/c mice in vivo. One day following implantation of the infected silicone tubes adjacent to the hind limb muscle tissue, the co-assemblies were administered via local injection at the incision sites at a dose of 250 mg/kg. The treatment was subsequently exposed to an alternating magnetic field (AMF) for 30 min. On day four, the animals were euthanized at the conclusion of the experimental period. Therapeutic outcomes were evaluated through histopathological and scanning electron microscopy (SEM) analyses, serum cytokine quantification, and colony-forming unit (CFU) assays. The drug-loaded co-assemblies demonstrated efficient payload release, resulting in thorough eradication of pathogenic biofilms, synergistic bactericidal activity, and effective mitigation of host tissue damage and inflammatory responses. Also in 2020, Cui et al. designed a near-infrared (NIR) light-driven, gold-asymmetrically functionalized mesoporous silica half-shell motor (HSM) loaded with vancomycin (HSMV), capable of penetrating biofilms and exploiting the synergistic effects of photothermal therapy and antibiotic treatment for the eradication of *Staphylococcus aureus* biofilms [92]. The therapeutic performance of this platform was evaluated using medical catheters implanted subcutaneously in the inner thigh of Wistar mice in vivo. Following catheter implantation, HSMV was administered via subcutaneous injection at a concentration of 1 mg/mL and a volume of 200 μL, and subsequently irradiated with a 650 nm laser (1.5 W/cm^2^) for 10 min. All experimental animals were monitored using an infrared camera and digital imaging. After seven days, the animals were euthanized, and biofilm eradication was assessed by scanning electron microscopy (SEM) and colony-counting assays. In addition, tissue samples from the implantation site, as well as the heart, liver, spleen, lungs, and kidneys, were harvested for histopathological evaluation. The HSMV-treated group exhibited only sparse bacterial presence and no detectable biofilm formation on the catheters, indicating efficient biofilm disruption and eradication of embedded bacteria compared with the control groups. Moreover, no significant histopathological abnormalities were observed in the major organs examined, suggesting favorable therapeutic biosafety.

Both of the aforementioned studies employed specifically engineered and fabricated nanorobotic systems for the targeted eradication of established biofilms. In contrast, in 2024, Quan et al. proposed a prophylactic coating strategy for titanium-based indwelling devices, utilizing composited silk fibroins (SFMA) integrated with Fe_3_O_4_ nanoparticles to enable on-demand, magnetically responsive disruption of methicillin-resistant *Staphylococcus aureus* (MRSA) biofilms [93]. This approach employed an externally applied NdFeB magnet to mechanically dislodge Fe_3_O_4_ nanoparticles, thereby synergistically enhancing the efficacy of conventional antibiotic therapy in a Sprague–Dawley rat model in vivo. Two days after subcutaneous implantation of infected Fe_3_O_4_/SFMA-coated titanium substrates, animals received vancomycin treatment (120 mg/kg) in combination with magnetic actuation to induce nanoparticle detachment. This combined intervention resulted in a two orders of magnitude reduction in bacterial burden within three days compared to the control. A subsequent administration of vancomycin together with additional magnetic disruption on day three produced a further two orders of magnitude decrease in bacterial counts by day seven. Therapeutic outcomes were evaluated through colony-forming unit (CFU) enumeration, scanning electron microscopy (SEM), and histopathological analyses using hematoxylin and eosin (H&E) and Masson staining. Biosafety assessment, including histological examination and blood biochemical analysis, revealed no observable adverse effects in the treated animals.

### 4.2. Eradication of Oral Biofilm-Associated Infections In Vivo

Dental biofilms are the primary etiological factor in diseases affecting the teeth and their supporting structures, notably dental caries and periodontal diseases [94]. Untreated caries in both permanent and deciduous dentitions remains among the most prevalent health conditions worldwide; in 2010, it was estimated to affect approximately 2.4 billion adults and 621 million children [95]. Although less prevalent than dental caries, severe periodontitis also ranks among the most widespread global health conditions, affecting approximately 11% of the world’s population in 2010 [96]. It is estimated that between 500 and 600 bacterial species colonize the oral cavity, contributing to the complexity and resilience of dental biofilms [97]. Despite the largely preventable nature of dental -biofilm-associated diseases through the maintenance of adequate oral hygiene, the global economic burden associated with their treatment remains substantial [98]. Moreover, oral biofilm associated bacteria and their metabolic byproducts have been implicated in the pathogenesis of several systemic conditions, including cardiovascular disease, chronic kidney disease, pulmonary disorders, diabetes mellitus, prostate, colon, and pancreatic cancers, preterm birth, erectile dysfunction, Alzheimer’s disease, and rheumatoid arthritis [99]. In 2019, Naha et al. reported the development of dextran-coated iron oxide nanoparticles, termed nanozymes (Dex-NZM), designed to selectively target oral biofilms and prevent severe dental caries in 15-day-old female Sprague–Dawley rats in vivo [100]. These nanozymes exploit the intrinsic peroxidase-like activity of iron oxide nanoparticles under acidic conditions. The dextran coating promotes incorporation into the exopolysaccharide (EPS) matrix of the biofilm, thereby enabling selective binding within the biofilm while minimizing adherence to adjacent gingival tissues. Upon topical administration, exogenously applied hydrogen peroxide (H_2_O_2_) is catalytically activated, resulting in localized bacterial eradication and degradation of the EPS matrix. Following oral inoculation with *Streptococcus mutans* UA159 and maintenance on a cariogenic diet, the animals underwent a 21-day treatment regimen. This protocol consisted of a 1 min topical application of Dex-NZM (1 mg/mL), immediately followed by exposure to H_2_O_2_ (1%, *v*/*v*). At the conclusion of the experimental period, the jaws were aseptically dissected and the teeth were harvested for caries assessment using Larson’s modification of the Keyes scoring system. Gingival and palatal tissues were collected for histopathological evaluation via hematoxylin and eosin staining, and biofilm samples were subjected to microbiological and microbiome analyses. The nanozyme-based topical therapy significantly reduced both the incidence and severity of carious lesions compared with control groups. Histological and microbiome analyses demonstrated no detectable adverse effects on surrounding host tissues or on the overall diversity of the oral microbiota. In 2021, Sun et al. reported the development of an oxygen self-generating nanocomposite designed to enhance selective antibacterial activity against anaerobe-induced periodontal disease in a male Wistar rat model in vivo [101]. The nanocomposite, designated F@Ce6-M NCs, comprised Fe_3_O_4_ nanoparticles co-encapsulated with chlorin e6 (Ce6) and coumarin 6 within an amphiphilic silane matrix, and subsequently modified with a MnO_2_ nanolayer. The MnO_2_ coating facilitated localized oxygen generation, thereby increasing oxygen availability within periodontal pockets to suppress anaerobic bacterial growth and enhance reactive oxygen species (ROS) production, ultimately improving biofilm eradication efficacy. Periodontal disease was induced by subgingival injection of a mixed bacterial suspension at the mandibular incisor site for seven consecutive days. Thereafter, 0.2 mL of F@Ce6-M NCs was administered locally under light irradiation (630–720 nm, 50 mW·cm^−2^, 3 min per day) for an additional three days. Post-treatment ROS levels were assessed via in vivo fluorescence imaging. Anaerobic bacterial burden was quantified through colony-forming unit counts, and the gingival tissues surrounding the infected sites were harvested for histopathological examination using hematoxylin and eosin staining. The F@Ce6-M NCs demonstrated significantly enhanced antibacterial efficacy and pronounced anti-inflammatory effects in vivo.

### 4.3. Eradication of Diabetic Wound-Associated Biofilm Infections In Vivo

Diabetes mellitus is widely recognized as a major public health concern, affecting an estimated 462 million individuals globally [102]. It imposes a substantial economic burden on healthcare systems, with patient care costs estimated to be at least 3.2 times higher than the average per capita healthcare expenditure, increasing to as much as 9.4 times in the presence of complications [103]. Among these complications, foot ulceration is the most commonly observed, with a reported lifetime incidence ranging from 19% to 34% [104]. This type of wound is characterized by persistent inflammation, reduced angiogenesis, and impaired cellular responses in key cell types such as macrophages and fibroblasts, leading to a chronic condition with often suboptimal healing outcomes [105]. In 2025, Deng et al. reported the development of a near-infrared (NIR)–responsive nanorobotic system constructed through the co-loading of indocyanine green (ICG) and lysostaphin onto spinous yolk–shell C/SiO_2_@C nanoparticles, designed to facilitate the eradication of methicillin-resistant *Staphylococcus aureus* (MRSA) biofilms and enhance wound healing in diabetic C57 murine models in vivo [106]. Diabetes was induced in the mice via intraperitoneal administration of streptozotocin (STZ) over five consecutive days. One week later, full-thickness wounds were created on the dorsal region and subsequently inoculated with methicillin-resistant *Staphylococcus aureus* (MRSA) to permit biofilm formation over the following two days. Subsequently, the mice received topical treatment and were exposed to near-infrared (NIR) light for 10 min daily over three consecutive days. Leveraging the synergistic effects of NIR-enhanced biofilm penetration, reactive oxygen species (ROS) generation by ICG, and lysostaphin-mediated bacterial cell membrane hydrolysis, the antibacterial efficacy reached 99%. Moreover, by day 16 of the experiment, the wounds were nearly completely healed, in contrast to the control groups, which exhibited either deterioration or only minimal improvement.

### 4.4. Discussion

The presented studies consistently report successful biofilm eradication in in vivo settings, representing an important step toward the translational advancement of the micro/nanorobotics field. Although it remains premature to draw definitive conclusions regarding their clinical applicability, several aspects of these studies suggest a greater translational relevance compared with the previously discussed studies in the section on antimicrobial strategies. In particular, a higher degree of methodological consistency can be observed across the reported studies, including the frequent use of colony-forming unit (CFU) quantification, histopathological evaluation of treatment outcomes, and clinical monitoring of therapeutic efficacy. This trend represents a substantial improvement over the heterogeneity in outcome assessment observed in earlier studies. Furthermore, greater emphasis has been placed on biosafety and toxicity evaluation, including analyses of serum cytokine levels and histopathological examination of distant vital organs. Such assessments constitute an essential component in the progression of these systems toward subsequent stages of preclinical and clinical development.

Nevertheless, several common limitations should be considered when evaluating the translational potential of these studies. Presented investigations have been conducted in small animal models over relatively short experimental periods and employ artificially induced acute infections, which do not adequately reproduce the chronic, polymicrobial, and immune-modulated characteristics of human biofilm-associated diseases. Consequently, the reported high eradication efficiencies and rapid tissue recovery may overestimate therapeutic performance under clinically relevant conditions. In addition, although all studies report successful biofilm eradication, the majority rely primarily on endpoint analyses, providing limited insight into bacterial persistence and the potential for biofilm regrowth. Future investigations should therefore incorporate long-term relapse studies together with real-time in vivo monitoring of biofilm dynamics to provide a more comprehensive evaluation of therapeutic efficacy. Addressing these limitations will be essential for advancing micro/nanorobot-based approaches from proof-of-concept investigations toward clinically applicable therapies for biofilm-associated infections.

**Table 3:** Overview of the micro/nanorobotic systems discussed for in vivo antibiofilm applications.

Micro/Nanorobots	Antimicrobial Strategy	Model	Biofilm	Administration Site	Target Site	Dose	Duration	Results	Ref.
Dual drug-loaded co-assemblies of heterogeneous mesoporous silica nanoparticles	Synergistic cargo delivery of AMP melittin and ATB ofloxacin	Male BALB/c mice	*Pseudomonas aeruginosa*	Local injection at the implantation site	Biofilm-infected silicone tube implanted adjacent to the hind limb muscle tissue	250 mg/kg	Three days	Biofilm eradication with minimized tissue damage, inflammation, and successful implantation	[91]
Gold asymmetrically functionalized and drug-loaded mesoporous silica half-shell motor	Synergistic photothermal and vancomycin therapy	Wistar mice	*Staphylococcus aureus*	Subcutaneous injection at the implantation site	Biofilm-infected medical catheter implanted subcutaneously in the inner thigh	2 mg/kg	Seven days	Biofilm eradication with no abnormalities in the major organs	[92]
Composited silk fibroins integrated with Fe_3_O_4_ nanoparticles as prophylactic surface coating	Synergistic mechanical detachment and local vancomycin therapy	Male Sprague–Dawley rat	Methicillin-resistant *Staphylococcus aureus*	-	Subcutaneously implanted biofilm-infected Fe_3_O_4_/SFMA-coated titanium disk	-	Seven days	Four orders of magnitude decrease in bacterial counts	[93]
Dextran-coated iron oxide nanoparticles	Reactive oxygen species generation	Female Sprague–Dawley rats	*Streptococcus mutans*	Topical application	Dental biofilm	1 mg/mL	Twenty-one days	Significant reduction in both the incidence and severity of carious lesions	[100]
Co-encapsulated Fe_3_O_4_, Ce6, and C6 in amphiphilic silane modified by MnO_2_ nanolayer	Reactive oxygen species generation	Male Wistar rat	*Streptococcus gordonii*, *Porphyromonas gingivalis*, *Fusobacterium nucleatum*	Local injection at the infected site	Previously infected cervical margin of the anterior mandibular teeth	0.2 mL	Three days	Significant antibacterial efficacy and pronounced anti-inflammatory effects	[101]
Co-loaded indocyanine green and lysostaphin onto spinous yolk–shell	Synergistic ROS generation and cargo delivery of AMP lysostaphin	Diabetic C57 mice	Methicillin-resistant *Staphylococcus aureus*	Topical application	Previously infected full-thickness wound on the dorsal region	5 mg/kg	Sixteen days	Antibacterial efficacy of 99% and nearly complete wound healing by the sixteenth day	[106]

## 5. Conclusions and Future Perspective

In this review, we summarize the intrinsic characteristics of bacterial biofilms and their implications for therapeutic limitations. Furthermore, the principles underlying antimicrobial mechanisms and the strategies employed by micro/nanorobots are discussed to highlight the breadth of approaches currently being explored. Finally, the translation of validated in vitro concepts into in vivo applications is examined.

Biofilm-associated infections represent highly challenging pathological conditions that contribute to increased morbidity and mortality among affected patient populations and necessitate complex therapeutic interventions, thereby imposing a substantial financial burden on healthcare systems worldwide. Additionally, the increasing antibiotic resistance, partially facilitated by conventional antibiotic therapies, threatens our future ability to effectively manage these diseases. Advances in nanoscience have led to the proposal of micro/nanorobots as potential solutions.

These micro/nanoscale structures are potentially capable of precise actuation, enabling them to selectively target the site of infection and mechanically disrupt the protective functions of the extracellular polymeric substances (EPS). By exploiting the extensive optimization potential for specific applications, such as functioning as cargo delivery systems for antibiotics, metals, antimicrobial peptides, and bacteriophages, or serving as platforms for the generation of reactive oxygen and nitrogen species (RONS), micro/nanoscale devices can facilitate bacterial cell death. This innovative strategy also holds promise for reducing antibiotic resistance.

Although the effectiveness of micro/nanorobots for bacterial eradication has been extensively investigated in recent years, the majority of studies have been conducted in vitro. While the findings of previous investigations are promising, they critically overlook key factors relevant to clinical translation, including pharmacokinetics, toxicity, immune responses, and the complex pathophysiological environment of in vivo infections, which contrasts markedly with the controlled conditions of in vitro experiments. To facilitate the translation of these findings into clinical practice, future in vitro research should prioritize the standardization of outcome measurements to enable more consistent and reliable comparisons across reported studies. Moreover, a greater emphasis on rigorous in vivo evaluation of the safety and therapeutic viability of these systems, while maintaining comparable methodological standardization, will be essential for advancing toward subsequent stages of clinical translation.

If future research successfully addresses the aforementioned limitations and considerations, these intelligent micro/nanorobotic systems may possess substantial potential to significantly influence clinical medical practice, particularly in the management of chronic and persistent infections that remain difficult to treat using conventional therapeutic approaches. In particular, the treatment of chronic biofilm-associated diabetic wounds represents a promising future application of micro/nanorobotic systems, as these conditions constitute a major therapeutic challenge and are frequently associated with prolonged healing and recurrent infection. Nanorobots employing synergistic strategies that combine reactive oxygen and nitrogen species (RONS) generation with mechanical disruption of biofilm structures may provide highly effective eradication of persistent and often antibiotic-resistant bacterial populations. Furthermore, the direct accessibility of wound environments enables localized administration of these systems, thereby reducing the need for systemic delivery and potentially minimizing adverse effects, including off-target oxidative stress and damage to healthy tissues in other regions of the body. This advantage of localized therapeutic activity may also be exploited in clinical dentistry. In this context, RONS-generating micro/nanorobotic systems could actively navigate the complex apical ramifications and inaccessible regions of root canal systems during the final stages of endodontic treatment, thereby facilitating more effective eradication of residual biofilms and improving overall treatment outcomes. Furthermore, following canal obturation, these systems could potentially remain confined within the sealed apical ramifications, thereby avoiding any systemic exposure. Cargo-delivery micro/nanorobotic systems may also represent a promising therapeutic approach in intensive care settings for the treatment of severe pneumonia. Inhalational administration of such systems could enable targeted and prolonged retention of antimicrobial agents within infected pulmonary tissues through enhanced controllability and localized delivery. This strategy may improve local therapeutic efficacy while reducing the reliance on conventional systemic antibiotic administration, thereby potentially minimizing additional toxic burden on vital organs, particularly the kidneys and liver. Finally, intelligent nanorobotic coatings prefabricated onto the surfaces of indwelling medical devices may substantially reduce the clinical burden associated with device-related infections. In the event of biofilm formation, these systems could be externally actuated to mechanically disrupt the biofilm matrix, thereby enhancing the efficacy of conventional antibiotic therapy against embedded microorganisms. Such an approach may improve infection control while reducing the likelihood of device failure and the subsequent need for surgical removal or replacement of the implanted device.

## Data Availability

No new data were created or analyzed in this study.
